# Blessing or curse: how the epigenetic resolution of host-transposable element conflicts shapes their evolutionary dynamics

**DOI:** 10.1098/rspb.2023.2775

**Published:** 2024-04-10

**Authors:** Yuheng Huang, Yuh Chwen G. Lee

**Affiliations:** Department of Ecology and Evolutionary Biology, University of California, Irvine, USA

**Keywords:** transposable elements, epigenetic silencing, evolutionary dynamics, stable equilibrium, ectopic recombination, epistasis

## Abstract

Transposable elements (TEs) are selfish genetic elements whose antagonistic interactions with hosts represent a common genetic conflict in eukaryotes. To resolve this conflict, hosts have widely adopted epigenetic silencing that deposits repressive marks at TEs. However, this mechanism is imperfect and fails to fully halt TE replication. Furthermore, TE epigenetic silencing can inadvertently spread repressive marks to adjacent functional sequences, a phenomenon considered a ‘curse’ of this conflict resolution. Here, we used forward simulations to explore how TE epigenetic silencing and its harmful side effects shape the evolutionary dynamics of TEs and their hosts. Our findings reveal that epigenetic silencing allows TEs and their hosts to stably coexist under a wide range of conditions, because the underlying molecular mechanisms give rise to copy-number dependency of the strength of TE silencing. Interestingly, contrary to intuitive expectations that TE epigenetic silencing should evolve to be as strong as possible, we found a selective benefit for modifier alleles that weaken TE silencing under biologically feasible conditions. These results reveal that the dual nature of TE epigenetic silencing, with both positive and negative effects, complicates its evolutionary trajectory and makes it challenging to determine whether TE epigenetic silencing is a ‘blessing’ or a ‘curse’.

## 1. Introduction

Transposable elements (TEs) are selfish genetic elements that copy and insert themselves into new genomic locations. They have been found in nearly all eukaryotes and can occupy a substantial portion of host genomes [1]. The prevalence and abundance of TEs are mainly driven by their ability to replicate faster than their host genomes, rather than any beneficial role they may have for organisms. In fact, TEs are often ‘harmful’. For instance, TEs can disrupt functions when inserted into essential genes [2,3] or provide ectopic regulatory sequences [4]. Recombination between non-homologous TEs, known as ‘ectopic recombination’, can lead to highly deleterious chromosomal rearrangements [5,6]. Furthermore, TEs have been found to alter the epigenome [7] and three-dimensional genome structures [8,9]. The replication of TEs to maximize their transmissions to the next generation thus comes at the expense of host genome function and, consequentially, organismal fitness. This antagonistic interaction between TEs and their hosts represents one of the most recognized and widespread intragenomic conflicts in eukaryotes [10].

One mechanism crucial to the resolution of intragenomic conflicts between TEs and their eukaryotic hosts is epigenetic silencing, typically mediated by small interfering RNAs (siRNAs) in plants or PIWI-interacting RNAs (piRNAs) in animals [7,11]. These small RNAs guide RNA interference (RNAi) protein complexes to TEs with complementary sequences, recruit histone-modifying enzymes and/or DNA methyltransferases, and lead to the deposition of repressive epigenetic modifications, such as DNA or histone methylation, at TE sequences [12,13]. Such epigenetic changes prevent the transcription of TEs, ultimately reducing their replication. Interestingly, TE-targeting siRNAs are the direct product of double-stranded RNAs converted from TE transcripts in flowering plants [14], while TE transcripts are part of the positive feedback loop that generates and amplifies piRNAs in metazoans [15]. Accordingly, the production of small RNAs, which serve as guides for the host cellular machinery to target TEs, relies on TEs themselves across systems.

However, the epigenetic silencing of TEs does not fully resolve the host–TE conflicts in the hosts' favour. Even in the presence of epigenetic silencing and other host-mediated mechanisms that suppress TE activity, many TE families remain active in the germline, with an estimated replication rate between 10^−5^ and 10^−4^ per copy per generation [16–19]. The same set of studies found that the rates at which TEs are naturally excised are lower than the replicative transposition of TEs, suggesting a non-zero net rate of TE accumulation even with host-mediated epigenetic silencing of TEs. Even worse, repressive epigenetic modifications enriched at silenced euchromatic TEs can ‘spread’ from TEs into adjacent sequences, including protein-coding genes (reviewed in [7]). This inadvertent side effect of TE epigenetic silencing is prevalent in genomes [20,21], between individuals [22,23], and across species [24,25]. How this spreading of repressive marks influences genome function has yet to be resolved (reviewed in [7,26]), but population genomic analyses have found stronger selection against TEs leading to such side effects [20,21,24,25], suggesting that this spreading effect is often harmful to organisms. The ineffectiveness of host-mediated epigenetic silencing in fully suppressing TE activities, along with the associated inadvertent harmful spreading of repressive marks, raises the question: *Is epigenetic silencing of TEs a blessing or a curse for the hosts?*

Previous verbal models proposed that the inadvertent ‘curse’ of TE epigenetic silencing may play a critical role in achieving stable containment of TEs in host populations [7,21,27]. Classic population genetic theory suggests that TE copy number in panmictic host populations is determined by the rates of TE replication (transposition) and TE removal (by excision or selection) [28,29]. When these two opposing forces balance each other, TE copy number reaches an equilibrium. Furthermore, whether this equilibrium of TE copy number is resistant to minor disturbance and can readily revert back to its equilibrium state, or is ‘stable’, depends on the relationship between host fitness and TE copy number ([28,29], reviewed in [7,27]). Specifically, with a constant rate of producing new TEs, a stable equilibrium of TE copy number can be achieved if each new copy has increasingly harmful effects, so that selection is more likely to remove them. Indeed, empirical evidence has demonstrated the existence of such ‘synergistic epistasis' of TEs’ harmful effects ([30], but see [31]). Because the epigenetic silencing of TEs is initiated by small RNA [7,11], whose generation depends on TE transcripts [14,15], it has been predicted that the *likelihood* of a TE being epigenetically silenced depends on TE copy number. Accordingly, the *number* of epigenetically silenced TEs is expected to increase with the square of TE copy number [7]. If so, even if the adverse effects of each *epigenetically silenced* TE remain constant, host fitness is expected to decline synergistically with the increase in the *total number of TE copies* in the host genome, fulfilling the requirement of stable containment of TE copy number [7].

Furthermore, with epigenetic silencing, the rates of producing new TE copies may not be constant, but decline with an increase in TE copy number. This copy-number-dependent transposition is the other mechanism proposed to drive stable equilibrium of TE copy number [28,29], even in the absence of selection against TEs. However, this explanation for TE containment has been less favoured (reviewed in [16,26]), because rates of TE transposition would have to be brought down by several orders of magnitude to match excision rates, which is not supported by empirical observations [16–18]. While epigenetic silencing of TEs holds the potential to contribute to copy-number-dependent rates of TE replication and stronger selection against TEs, its predicted role in driving stable containment of TE copy numbers remains unexplored.

Whether the epigenetic silencing of TEs is a blessing or curse hinges upon whether the benefits of reducing TE replication outweigh the costs arising from the inadvertent spreading of repressive marks. Interestingly, this balance appears to vary between species, as evidenced by the observed difference in the extent of this spreading effect in closely related species [24,25] and across taxa (reviewed in [7]). Modifier alleles that enhance TE silencing may be favoured by natural selection owing to reduced rates of TE replication, but they may also face stronger negative selection resulting from the more extensive harmful spreading of repressive marks. Conversely, modifier alleles that weaken TE silencing may have the immediate selective benefit of alleviating the harmful side effects of TEs, but they can also drive the accumulation of TEs owing to the increased rates of TE replication. The dual roles of modifier alleles in altering the rates of TE transposition and the strength of selection against TEs make it challenging to predict whether TE epigenetic silencing would evolve stronger or weaker.

In this study, we performed forward simulations to study how copy-number-dependent epigenetic silencing of TEs influences TE evolutionary dynamics, through either reduced transposition rate, selection against the associated spreading of repressive marks, or both. Focusing on conditions that allow TE copy numbers to reach a stable equilibrium, we explored the invasion dynamics of modifier alleles that alter TE epigenetic silencing, revealing another surprising ‘curse’ of this epigenetic resolution of host–TE conflicts.

## 2. Methods

### (a) Basic model for transposable element evolutionary dynamics

**Figure 1.**
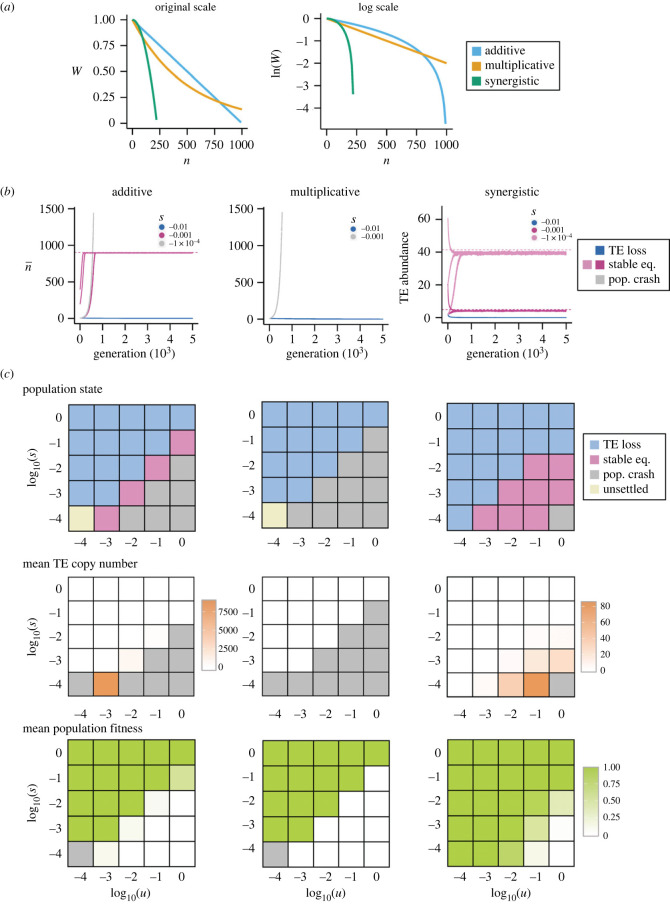
Evolutionary dynamics of transposable elements (TEs) vary under different modes of interactions of TEs' deleterious impacts in the absence of TE epigenetic silencing. (*a*) Fitness functions of TE copy number, *n*, under additive, multiplicative and synergistic fitness effects. To show all three fitness functions in one figure, we chose the following values of *s*: 10^−3^ for additive fitness effect, 2 × 10^−3^ for multiplicative fitness effect, and 2 × 10^−5^ for synergistic fitness effects. (*b*) Trajectories of mean TE copy number under three modes of the interaction of TEs’ fitness effects, with *u* = 0.01 and *s* = 10^−4^, 10^−3^ and 10^−2^. Purple, blue and grey indicate stable equilibrium of TE copy number, TE loss and population crash, respectively. Dashed lines indicate equilibrium TE numbers predicted by the analytical model for infinite population size [28]. (*c*) Heat maps showing population conditions (stable equilibrium of TE copy number, TE loss or population crash), mean TE copy number and mean population fitness for different *u* and *s*, when *r* = 10^−6^. Note that when *u* and *s* are both weak (10^−4^; *Nu* and *Ns* ≈ 1) for additive and multiplicative fitness effects, the impact of genetic drift is prominent and we were unable to determine the equilibrium condition (unsettled)*.* Results with *r* = 10^−7^ are consistent (electronic supplementary material, figure S1).

All simulations were performed in SLiM version 4.0.1 [32]. We started with SLiM's recipe for the evolutionary dynamics of TEs, which models a diploid population with constant population size (*N*) with separate sexes, random mating and discrete generations (Wright–Fisher model). At the beginning of a simulation, a fixed number of TEs (TE copy number, *n*) are randomly positioned onto one of the two homologous chromosomes. The fitness of an individual (*W*), which specifies the probability that an individual will be chosen as a parent for the next generation, is determined by three modes of interactions among TEs' fitness effects (figure 1*a*):

2.1 additive: W=1−s ∗ n,

2.2 $$\mathrm{multiplicative}:\;W={\left(1-s\right)}^{n}$$

2.3 $$\mathrm{and}\;\mathrm{synergistic}:\;W=1-s\;*\;n^{2},$$

where *s* determines the strength of selection against TEs. In the next generation, each TE makes a new copy that inserts into a random position in the genome, with transposition rate *u*. For one replicate of the simulation, this selection–reproduction–transposition cycle was repeated for a designated number of generations (see below). It is important to note that these parameters are *in the absence of* the effects of TE epigenetic silencing, and we assumed that negative fitness effects of TEs are caused by other mechanisms that generate deleterious but nonlethal/nonsterile mutations (e.g. insertion into functional sequences).

For each parameter setting, the simulation was run for 100 replicates and for at least 10^4^ generations, or until the population reached two *unstable* states: (1) TE loss, or the mean TE copy number reaches zero, or (2) population crash, or the mean fitness of a population declined to lower than 0.1%. A third possible outcome is that the TE copy number reaches stable equilibria, which we determined using two criteria. First, we visually examined whether the TE copy number reached a relatively stable value for at least 5000 generations. In addition, under a stable equilibrium, different replicates should reach a similar equilibrium copy number. Accordingly, we regarded a simulation with a set of parameters reaching stable equilibrium when the relative variance for TE copy number among replicates (variance divided by the mean) was less than 1/*Ns*, which takes into account the varying strength of selection (determined by *s*) relative to genetic drift (determined by *N*).

#### (i) Model parameters

Instead of parameterizing our simulations according to a specific organism, we explored a large parameter space and discuss the results in the context of various systems. We simulated 10^4^ diploid individuals (*N*), each of which has two 50 Mb chromosomes. Estimated *u* per copy per generation for TE families that have established in the population, instead of recently horizontally transferred, is around 10^−4^ across systems, including those with small (e.g. human; [33]) and large population sizes (e.g. *Drosophila* and *Daphnia* [17,18]). Nevertheless, documented historical bursts of TE activities (e.g. [34]) suggest that transposition rates of existing TE families may occasionally be higher than these estimates. Accordingly, we explored values of *u* between 10^−4^ and 1. The parameter *s* was modelled between 10^−^^4^ and 1, capturing both weak and strong selection. The simulated crossover rate (*r*) ranged between 10^−7^ and 10^−6^ bp^−1^, which covers intermediate to high estimated rates across systems [35]. See electronic supplementary material, table S1 for parameters and variables.

We are mainly interested in understanding how selection and transposition, when influenced by the epigenetic silencing of TEs, shape the evolutionary dynamics of TEs and host alleles. Interactions of these two forces with genetic drift are not the focus of this study. Accordingly, for a large part of the parameter space we explored, the products between population size and the strength of biological forces modelled are much greater than one (e.g. *Nu* and *Ns* ≫ 1). Under these conditions, the simulations are largely deterministic and mainly determined by the absolute value of the biological parameters. On the other hand, according to population genetic theories [36] and simulations modelling TEs [37], when compound parameters are around or smaller than one (e.g. *Nr* < 1), the evolutionary dynamics are governed by these compound parameters.

### (b) Models with transposable element epigenetic silencing

We modelled the epigenetic silencing of each TE as occurring after reproduction and re-established every generation. With the assumption that the epigenetic silencing of one TE does not reduce the probability of another TE being silenced by small RNAs (i.e. small RNAs are in excess), the probability of a TE being silenced should depend on total small RNA abundance, which further depends on TE copy number [7]. Accordingly, we modelled the magnitude of TE epigenetic silencing (*M*) as a Poisson distribution with mean *an*, where *a* is set as 0.02. The selection coefficient and transposition rate in the presence of TE epigenetic silencing ($s_{i}^{\prime }$ and $u_{i}^{\prime }$, respectively) are modelled as

2.4 $$s_{i}^{\prime }=s_{i}\;*\;(1+bM_{i})\;$$

and

2.5 $$u_{i}^{\prime }=u_{i}\;*\;(1-kM_{i}),$$

where the subscript *i* indicates the *i*th TE. Equation (2.4) assumes that the selection against the spreading of repressive marks from silenced TEs is *in addition* to that of other deleterious, but non-lethal/sterile, effects of TEs. We explored three values for *b*: 0.2 (selection against silencing is weaker than or of a similar order of magnitude to that for other harmful effects of TEs), 2 and 20 (selection against silencing is strong and dominates). We set *k* = 0.2, and negative $u_{i}^{\prime }$ was assigned a value of zero.

The fitness of an individual with additive or multiplicative fitness effects with *epigenetically silenced* TEs becomes:

2.6 $$\mathrm{additive}:\;W=1-\sum_{i}^{n}s_{i}^{\prime }$$

and

2.7 $$\mathrm{multiplicative}:\;W=\prod_{i}^{n}\left(1-s_{i}^{\prime }\right).$$

It is worth noting that we focused on how epigenetic silencing of *existing* TE families influences the *equilibrium* states of TE evolutionary dynamics, instead of the invasion dynamics of recently horizontally transferred TE families, which was addressed in other studies [38,39].

### (c) Comparison of simulation results and analytical solutions for equilibrium transposable element copy number

According to eq. 19b in [28], we derived analytical solutions for equilibrium TE copy numbers under infinite population sizes in the absence and presence of impacts of TE epigenetic silencing (detailed in electronic supplementary material, text S1 and text S2). Our simulation results are largely consistent with analytical predictions, which are included in electronic supplementary material, tables S2 and S3 and discussed in text S1. When TE epigenetic silencing impacts both selection and transposition, the derivation of equilibrium TE copy number requires solving cubic equations, which is beyond the scope of the current study. Accordingly, we mainly present results from stochastic simulations in the main text.

### (d) Models for the evolution of transposable element silencing

To investigate the invasion dynamics of modifier alleles enhancing or weakening TE epigenetic silencing, we simulated when *u =* 0.01 and *s =* 10^−3^ under three scenarios: TE epigenetic silencing impacting selection (*b* = 0.2), impacting transposition (*k* = 0.2) or impacting both (*b* = 0.2 and *k* = 0.2). We first simulated the population *without* the modifier alleles for 5000 generations to allow TE copy number to reach equilibrium (pre-invasion phase). At generation 5001, or generation 1 of the invasion phase, one modifier locus was positioned in the middle of the chromosome in randomly sampled individuals to achieve a population frequency of 10%. A modifier allele enhancing TE silencing leads to the increase of *a*, which determines the mean strength of TE epigenetic silencing, with

2.8 $$a^{\prime }=a\;*\;(1+0.5\;*\;H),$$

where *H* equals 1 for homozygotes of the modifier allele and 0.5 for heterozygotes. For modifier alleles weakening TE silencing,

2.9 $$a^{\prime }=a\;*\;(1-0.5\;*\;H).$$

We investigated whether the inclusion of ectopic recombination, which is expected to generate dominant lethal or sterile mutations [5,40] and may be limited by epigenetic silencing of TEs [7,41], influences the invasion dynamics of modifier alleles for TE silencing. Because the rates of ectopic recombination significantly reduce when transgenes are in homozygous states [42], we modelled ectopic recombination happening between pairs of *heterozygous* TEs with rates ranging from 10^−5^ to 10^−3^. Individuals with any ectopic recombination between TEs were set to have fitness zero. TE epigenetic silencing was set to either have no effect on ectopic recombination or completely suppress it when *M* is greater than a threshold (1.5 for *u* = 0.1, 5 for *u* = 0.01 and 0.001).

## 3. Results

### (a) Stable equilibrium of transposable element copy number is rarely reached in the absence of synergistic epistasis

A goal of this study is to investigate whether TE epigenetic silencing, which was predicted to result in synergistic fitness effects [7], can indeed drive stable containment of TE copy number. To set the basis for our following investigation, we first performed simulations under three modes of interaction of TEs’ fitness effects *in the absence of TE epigenetic silencing* (figure 1*a*). Under 25 combinations of *u* and *s*, we found that TE copy number rarely reaches stable equilibria in the absence of synergistic epistasis. With additive fitness effects, stable equilibria (see Methods) occur only when *s* is roughly an order of magnitude smaller than *u* (figure 1*b,c*, left for *r* = 10^−6^; electronic supplementary material, figure S1 for *r* = 10^−7^; and electronic supplementary material, table S2 for numerical values and comparisons with analytical predictions), a result consistent with previous theoretical prediction [28] (see electronic supplementary material, text S1 and text S2 for details). When *s* is of a similar order of magnitude to *u* or higher, TEs are completely removed (TE loss), and when *s* is two orders of magnitude smaller than *u*, TE copy number increases indefinitely until the mean fitness of the host population becomes zero (population crash). Under multiplicative fitness effects, none of the simulations reaches stable equilibrium (figure 1*b,c*, middle; electronic supplementary material, figure S1 and table S2; also see electronic supplementary material, figure S2 for finer exploration of the parameter space), again consistent with previous theoretical prediction [28] (electronic supplementary material, text S2). By contrast, TE copy number reaches stable equilibrium under a wide range of conditions in the presence of synergistic epistasis (figure 1*c*, right; electronic supplementary material, figure S1). Overall, these results confirm theoretical predictions that stable containment of TE copy number is highly unlikely in the absence of synergistic epistasis [28], providing the basic models for our following analysis on the effects of TE epigenetic silencing.

### (b) Epigenetic silencing of transposable elements expands the conditions that allow stable containment of transposable element copy number

**Figure 2.**
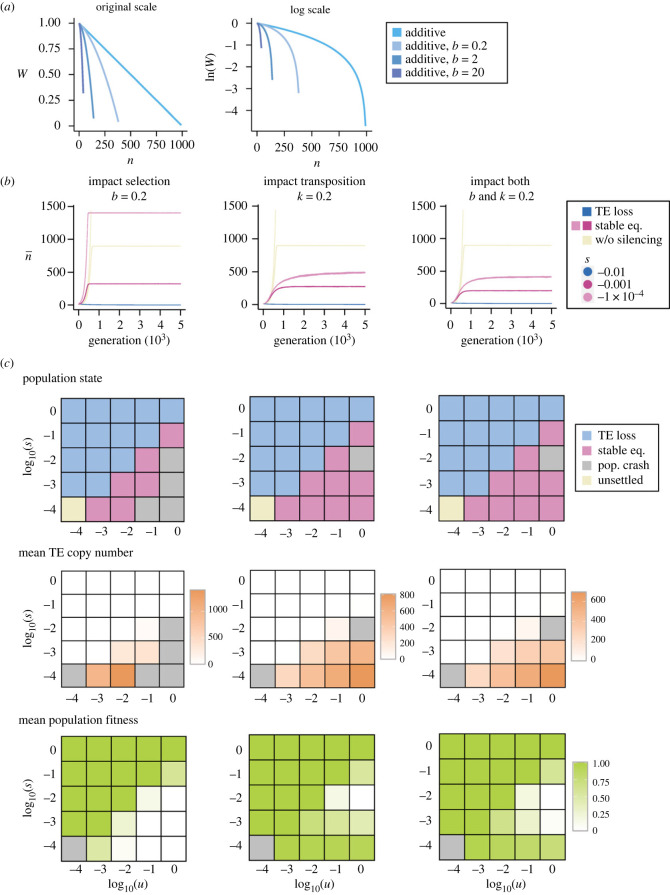
Transposable element (TE) epigenetic silencing influences the population dynamics of TEs. (*a*) Fitness functions *W* under additive fitness effects with and without the associated side effects of TE epigenetic silencing. (*b*) Trajectories of mean TE copy number under different functional consequences of TE epigenetic silencing: influencing only selection (left), influencing only transposition (middle) and influencing both (right). Trajectories for *u* = 0.01 and *s* = 10^−4^, 10^−3^ and 10^−2^ under *r* = 10^−6^ are shown. Purple and blue indicate stable equilibria of TE copy number, and TE loss in the presence of TE epigenetic silencing, while the yellow line indicates the trajectory in the absence of TE epigenetic silencing. (*c*) Heat maps showing population conditions (stable equilibrium of TE copy number, TE loss or population crash), mean TE copy number and mean population fitness for different *u* and *s*, when *r* = 10^−6^ and under three possible impacts of TE epigenetic silencing. Results with *r* = 10^−7^ are consistent (electronic supplementary material, figure S3).

We investigated how TE epigenetic silencing influences the conditions required for stable containment of TE copy number by impacting selection, transposition rates, or both. The spreading of repressive marks from epigenetically silenced TEs, which we refer to as ‘side effects of TE silencing’ hereafter, was shown to result in increased selection against TEs [20,21,24,25]. Because the strength of selection against such an effect has not been estimated, we modelled this process as being *in addition to* other harmful effects of TEs. We modelled the fitness effect of silencing as a linear function of the magnitude of TE epigenetic silencing, which in turn depends linearly on TE copy number (see Methods). Accordingly, even when the fitness of an individual is determined by the *sum* of the fitness effects of *silenced* TEs (equation (2.6) in Methods), which follows the definition of *additive* fitness effects, individual fitness would actually depend on the square of TE copy number and decrease faster than linearly with an increase in TE copy number (figure 2*a*), potentially meeting the theoretically predicted requirement for stable containment of TE copy number [28].

Our simulations showed that, when selection against the side effects of TE silencing and other harmful effects of TEs are of a similar order of magnitude (*b* = 0.2, see Methods), TE copy number reaches stable equilibrium even when *s* is more than an order of magnitude smaller than *u* (figure 2*b,c*, left for *r* = 10^−6^ and electronic supplementary material, figure S3 for *r* = 10^−7^)*,* which contrasts with the results in the absence of epigenetic silencing (figure 1*c*, left). The ratio between the *effective s* under equilibrium TE copy number (estimated using equation (2.4)) and *u* ranges between 0.025 to 0.5, significantly expanding conditions allowing stable containment of TEs (electronic supplementary material, table S3). In addition, the inclusion of fitness costs of TE silencing results in a lower equilibrium mean copy number and higher population mean fitness compared with simulations without this effect (figure 2*b*, left). Interestingly, the trajectories of TE copy number are similar to those under simple additive fitness effects (figure 1*b*, left versus figure 2*b*, left): TE copy number increases exponentially initially and rapidly slows down after surpassing a certain threshold, similar to a truncation selection scenario. This observation does not entirely align with the expectation that selection against the side effects of TE epigenetic silencing should lead to similar trajectories of TE copy number to those under synergistic fitness effects [7]. We thus further explored the population dynamics of TE copy number with increased relative strength of selection against such effects compared with other harmful effects of TEs (*b* = 2 and 20; see Methods). As the fitness cost of TE epigenetic silencing increases (i.e. *b* increases), the fitness function becomes more strongly downwardly curved (figure 2*a*), allowing the second derivatives of the function to meet the theoretically predicted requirement for stable TE containment (eq. 21b of [28]). Consistently, the trajectories of TE copy number become similar to those with synergistic fitness effects, and TE copy number reaches stable equilibria under a further expanded parameter space (electronic supplementary material, figures S4 and S5). These observations suggest that the side effect of TE epigenetic silencing, previously considered a ‘curse’, can reduce TE load, lead to stable containment of TE copy number, and have long-term benefits to host populations.

We also explored how the impacts of TE epigenetic silencing on transposition rate influence the conditions for stable containment of TE copy number. We modelled *changes* in transposition rate as a linear function of the magnitude of TE epigenetic silencing, itself a linear function of TE copy number (*k* = 0.2; see Methods). By allowing TE silencing to reduce transposition, TE copy number again reaches stable equilibrium with significantly expanded conditions compared with additive fitness effects alone (figure 1*c* versus figure 2*c*, middle; electronic supplementary material, figure S3). The ratio between *s* and *effective u* (estimated using equation (2.5)) that allows stable equilibria ranges between 0.1 to 1.25 (electronic supplementary material, table S3), suggesting that stable containment of TE copy number is possible even when *s* is higher than *effective u*. Curiously, the trajectories of TE copy number are similar to those with synergistic fitness effects (figure 2*b*, middle versus figure 1*b*, right). The simulations incorporating the impacts of TE epigenetic silencing on both selection and transposition rate (*b* > 0 and *k* = 0.2) behave as expected, with an expanded parameter space allowing stable containment of TEs, even when compared with epigenetic silencing influencing only the selection process (figure 2*b*,*c*; electronic supplementary material, figure S3 for *b* = 0.2, and electronic supplementary material, figure S5 for *b* = 2 and 20; electronic supplementary material, table S3). In similar investigations involving multiplicative fitness effect, where TE copy number failed to reach stable equilibrium under any parameter settings in the absence of TE epigenetic silencing (figure 1), we also observed TE copy number can be stably contained for some parameters when incorporating the effects of TE epigenetic silencing (electronic supplementary material, figure S6 and table S4).

### (c) Modifier alleles that weaken the epigenetic silencing of transposable elements spread readily and jeopardize population mean fitness

To investigate how TE epigenetic silencing evolves, we studied whether and how a new allele modifying TE epigenetic silencing may spread in a population whose TE copy number has already reached a stable equilibrium. We focused on the evolutionary dynamics of modifier alleles under conditions where TE copy number reaches stable equilibrium for all three possible impacts of TE epigenetic silencing (*b* = 0.2, *k* = 0.2 and both = 0.2, figure 2*c*; see Methods), mainly *u* = 0.1, 0.01 and 0.001 while the corresponding *s* is an order of magnitude lower. Our following discussions primarily focus on when the fitness effects of TE epigenetic silencing are of comparable magnitude to that of other fitness effects (*b* = 0.2), which already leads to significant impacts on the evolutionary dynamics of modifier alleles (see below).

**Figure 3.**
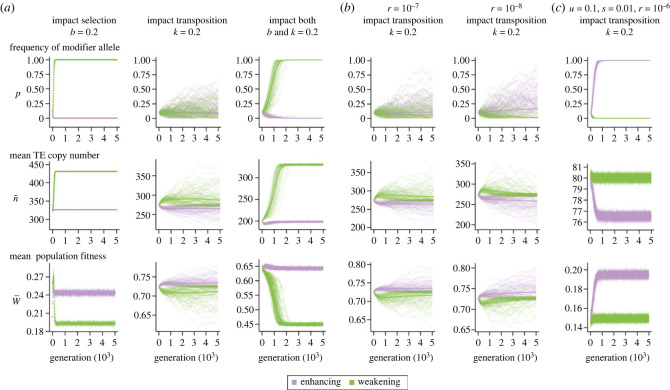
Population dynamics of modifier alleles that enhance or weaken transposable element (TE) epigenetic silencing. The trajectories for the frequencies of modifier alleles that enhance (purple) or weaken (green) TE silencing, TE copy number, and mean population fitness for 100 replicated simulations (thin lines) and their mean (thick line) *during the invasion phase* are shown. (*a*) The population dynamics of modifier alleles under *u* = 0.01, *s* = 0.001 and *r =* 10^−6^ when TE epigenetic silencing only impacts selection (*b* = 0.2), transposition (*k* = 0.2), or both (*b* and *k* = 0.2). (*b*) Dynamics of modifier alleles under *u* = 0.01, *s* = 0.001 with lower recombination rates (*r =* 10^−7^ and *r =* 10^−8^) and when TE silencing only impacts transposition (*k* = 0.2). See electronic supplementary material, table S5 summarizing results when *b* = 0.2 and *b* and *k* = 0.2. (*c*) Modifier alleles with *u* = 0.1, *s* = 0.01 and *r =* 10^−6^ and when TE silencing only impacts transposition (*k* = 0.2). See electronic supplementary material, table S5 for results when *b* = 0.2 and *b* and *k* = 0.2 and other recombination rates. Note that, for graphs of TE copy number and mean population fitness, *y*-axis does not start from zero.

We first investigated the evolutionary dynamics of modifier alleles under intermediate impacts of transposition and selection (*u* = 0.01, *s =* 0.001) and free recombination (*r* = 10^−6^). When TE silencing only influences selection (*b*= 0.2), modifier alleles *enhancing* TE silencing are rapidly removed from the population, which likely results from the increased fitness cost associated with the modifier alleles. On the contrary, modifier alleles *weakening* TE silencing quickly spread to fixation (figure 3*a*), leading to an accumulation of TEs and a reduction of the population mean fitness. Such a finding suggests that while a modifier weakening TE silencing may result in an immediate reduction in fitness costs associated with TE silencing and thus be selected for, in the long term, these alleles drive an increase in TE burden and compromise population fitness. When TE epigenetic silencing only influences TE transposition rate (*k* = 0.2), neither type of allele spreads through the population (figure 3*a*). Owing to the fact that the fitness benefit of reducing transposition rate has a minimal effect on the fate of the modifier allele (i.e. when *k* = 0.2), modifier alleles that affect both transposition rate and selection (*b* = 0.2 and *k* = 0.2) have a fate that is largely similar to that of alleles that affect only selection (figure 3*a*). A major difference is the slower initial spread of the weakening allele, which may have been driven by an initial increase of TE copy number with elevated transposition rate, and thus overall weaker selective advantage of the allele.

We further studied the evolutionary dynamics of modifier alleles under reduced rates of recombination (*r* = 10^−7^ and 10^−8^), which were theoretically predicted to influence the spread of alleles reducing TE transposition rate [43]. Interestingly, while reduced recombination rate has limited impacts on the evolutionary dynamics of modifier alleles when TE epigenetic silencing only influences selection (electronic supplementary material, table S5), modifier alleles enhancing silencing are now able to invade when TE epigenetic silencing only influences TE transposition rate (figure 3*b*). Nevertheless, when there is a cost for TE epigenetic silencing, modifier alleles enhancing silencing are still unable to spread (electronic supplementary material, table S5).

We also explored scenarios where the impacts of transposition and selection are strong (*u* = 0.1, *s =* 0.01) and found that enhancing alleles readily rise in frequency and go to fixation when epigenetic silencing *only* influences TE transposition (figure 3*c*). Yet, when there is a cost associated with TE silencing, selection still favours modifier alleles weakening silencing (electronic supplementary material, table S5). Neither modifier alleles enhancing nor those weakening TE silencing are able to spread under all conditions explored when the impacts of transposition and selection are both weak (*u* = 0.001, *s =* 10^−4^; electronic supplementary material, table S5), where the impacts of genetic drift might dominate. In summary, our findings indicate that, when there are fitness costs of TE epigenetic silencing, modifier alleles that enhance TE silencing cannot spread under any conditions explored. Conversely, modifier alleles that weaken TE silencing quickly become fixed in the population, despite their long-term detrimental impact on the fitness of the host populations, leading to another ‘cursed’ condition.

### (d) Potential suppressive effects of transposable element epigenetic silencing on ectopic recombination halt the spread of modifier alleles weakening transposable element epigenetic silencing

Previous studies established a link between the enrichment of repressive epigenetic marks and a decrease in the occurrence of double-stranded breaks, the first step of meiotic recombination [44,45]. Because the same repressive epigenetic marks are found at epigenetically silenced TEs, TE epigenetic silencing might suppress local recombination and prevent the occurrence of ectopic recombination between non-homologous TEs [7,41]. This predicted effect may hold particular significance for the evolution of modifier alleles of TE epigenetic silencing for several reasons. An appreciable frequency of TE-induced dominant lethal or sterile mutations was theoretically predicted to increase the selective benefits of alleles that suppress TE transposition rates [43], and mutations generated by ectopic recombination, such as chromosomal rearrangements, could have such fitness impacts [5,40]. Additionally, the potential inhibitory effects of TE epigenetic silencing on ectopic recombination may suggest a higher fitness cost associated with modifier alleles weakening TE silencing and have the potential to prevent the spread of such alleles.

**Figure 4.**
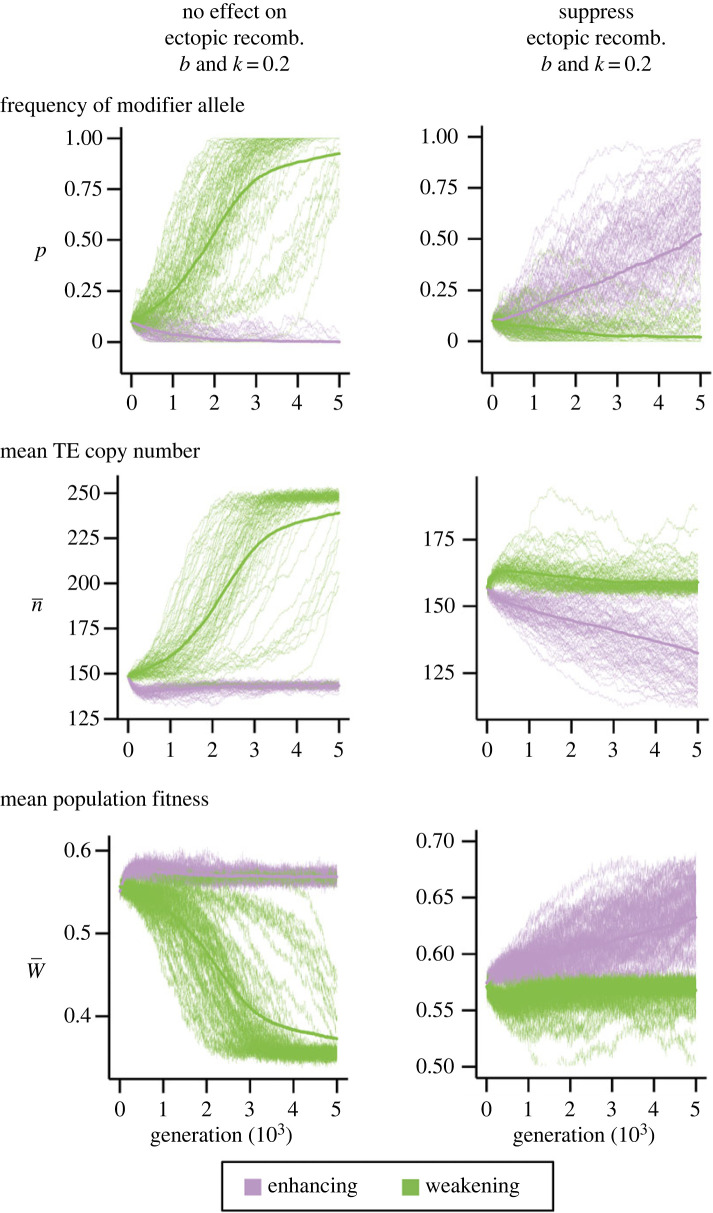
Population dynamics of modifier alleles that enhance or weaken transposable element (TE) epigenetic silencing in the presence of ectopic recombination. The trajectories for the frequencies of modifier alleles enhance (purple) or weaken (green) TE silencing, TE copy number and mean population fitness when TE epigenetic silencing has no effect on (*a*) or suppresses (*b*) ectopic recombination under *u* = 0.01, *s* = 0.001, *r =* 10^−6^ and 10^−3^ rates of ectopic recombination. A hundred replicated simulations (thin lines) and their mean (thick line) are shown. See electronic supplementary material, table S6 for a summary of results under all conditions explored.

We investigated how ectopic recombination, which was modelled to occur between non-homologous TEs and lead to zero fitness of an individual (see Methods), influences the evolutionary dynamics of enhancing and weakening alleles when TE epigenetic silencing impacts both selection and transposition (*b* and *k* = 0.2). By assuming that the rates of ectopic recombination follow that of homologous recombination [42,46] and the fact that a TE is several kilobases in size, we explored a range of ectopic recombination rates (*r*_e_ ≈ 10^−5^–10^−3^). When the impacts of transposition and selection are intermediate (*u =* 0.01, *s* = 0.001) or weak (*u =* 0.001, *s* = 10^−4^), the inhibitory effects of TE epigenetic silencing on ectopic recombination radically changed the evolutionary dynamics of modifier alleles—enhancing allele spread, resulting in reduced TE abundance and higher population mean fitness (figure 4; *r*_e_ needs to be 10^−3^ for *u =* 0.01 and 10^−4^ for *u =* 0.001; see electronic supplementary material, table S6 for a summary of results under different parameter settings). In stark contrast, modifier alleles that weaken TE silencing no longer spread. The immediate fitness cost from increased frequencies of dominant lethal likely limits the spread of such alleles, ‘rescuing’ the population from the accumulations of TEs and reduction in population fitness. Interestingly, when the epigenetic silencing of TEs has no impact on ectopic recombination (figure 4), the population dynamics of modifier alleles are qualitatively similar to simulations without ectopic recombination (figure 3*a*). This observation suggests that, for enhancing alleles to rise in frequency, the direct benefit of suppressing ectopic recombination is needed to overcome the reduction in fitness due to enhanced silencing. It is worth noting that the rates of ectopic recombination need to be as high as reported above to allow the spread of enhancing alleles while restraining the rise in frequencies of weakening alleles (electronic supplementary material, table S6).

## 4. Discussion

This study was inspired by the observation that TE epigenetic silencing can suppress transposition rates [11], lead to the spreading of repressive marks from TEs to nearby functional regions [7], and potentially change the way selection acts on TEs directly [7]. Our simulations found that selection against the harmful side effects of TE epigenetic silencing allows the stable containment of TEs under a wide range of parameters owing to the predicted synergistic fitness effects arising from the intrinsic biological mechanism of TE silencing. Especially, when the selective cost of epigenetic silencing is strong and dominates other deleterious effects, the trajectories of TE copy number become similar to those under simple synergistic fitness effects, akin to classic models that focus on strong fitness impacts of TE-mediated ectopic recombination without considering other deleterious effects of TEs [40]. Empirical studies have suggested that the functional consequences of TE epigenetic silencing could indeed be strong (e.g. via alternation of three-dimensional genome structure; [8,9]), but may also be weak (e.g. via perturbing gene expression, [23,24]). However, we lack estimates for relative frequencies of strong and weak effects, and of their importance in determining individual fitness compared with other harmful effects of TEs. Interestingly, the impacts of TE silencing on regulated transpositions alone can lead to stable equilibria of TE copy number over an even wider parameter space than selection against the associated harmful side effects.

Our simulations assume that the strength of TEs being epigenetically silenced depends positively on TE copy number, an idea supported by empirical observations. The strength of the side effects of TE epigenetic silencing positively correlates with the total abundance of small RNA targeting a TE family [21,24,47], which typically positively associates with TE copy number [48,49]. In fact, positive correlations have been observed between TE copy number and the strength and/or side effects of TE epigenetic silencing [24,50]. However, there are clear exceptions to this assumed copy-number dependency, such as cases where only a few copies of TE insertions are able to suppress the transposition of entire TE families [51,52]. Additionally, the copy-number dependency of TE transposition rates could arise from mechanisms other than epigenetic silencing, such as small RNA-mediated post-transcriptional silencing [53,54] and the dominant-negative effects of transposases on TE replication [55,56]. These alternative mechanisms might similarly expand the biological conditions that allow stable containment of TE copy number.

The long-term benefits of TE epigenetic silencing in containing TE copy number invite the prediction that selection might favour alleles that strengthen silencing. Yet, our simulations suggest a more complex picture. Under most conditions explored, enhancing alleles are disfavoured. The underlying reason is that the fitness benefits of alleles enhancing silencing depend on their associations with the genome-wide altered TE load, which are easily broken by recombination [43]. Selection, instead, favours alleles weakening silencing, whose benefit is mediated through their immediate impact on reducing the harmful side effects of all TEs across the genome. The selectively favoured reduction in TE suppression would lead to the accumulation of TEs and create conditions that further promote the selection for modifier alleles weakening TE silencing, ending in a downward spiral of population fitness. Nevertheless, alleles enhancing TE silencing are favoured under several conditions—under high rates of transposition (approx. 0.1 per copy per generation) or strong genome-wide linkage (*Nr* ≈ 10^−4^) (also see Methods for conditions where TE evolutionary dynamics are largely determined by absolute values of biological parameters or their products with population sizes). While the rate of transposition needed is much higher than the estimates for extant populations [17–19,33], there may be a sufficient linkage between modifier alleles and reduced TE load to favour enhanced TE suppression in species with small population size and/or low recombination rate, such as mammals and certain plant species [35]. It is worth noting, though, that modifier alleles enhancing silencing are always disfavoured in the presence of fitness costs of TE silencing, a condition more biologically realistic with the widely documented inadvertent side effects of TE epigenetic silencing [7].

Then, why are TEs not more active in eukaryotes? Our simulations suggest that the potential suppressive impacts of TE silencing on TE-mediated ectopic recombination may provide a possible reason—modifier alleles enhancing TE silencing spread because these alleles are protected from dominant lethal mutations caused by ectopically recombined TEs. For this to occur, the rate of ectopic recombination needs to be high, ranging between 10^−4^ and 10^−3^ under the conditions we simulated. The available empirical evidence for the frequency of ectopic recombination being at least this high is equivocal, with estimates between 10^−8^ and 10^−6^ per copy per generation between transgenes [57,58] and 10^−5^ and 10^−4^ between non-homologous TEs [5]. In addition, TE epigenetic silencing may also suppress other sources of dominant lethal or sterile mutations, such as chromosomal breakage induced by TE activities [59,60], structural rearrangement generated by alternative transposition [61], or sterility arising from elevated TE activities [62,63]. Yet, we similarly lack estimates of their rates. Importantly, to resolve whether natural selection would overall favour modifier alleles enhancing or weakening TE epigenetic silencing requires more than just estimated rates of TE-mediated dominant lethal or sterile mutations—we need to confirm that TE epigenetic silencing suppresses these mutations and estimate the strength of this suppression [7,41].

Another route to decipher whether selection favours enhanced or weakened TE silencing is to investigate the functional effects of modifier alleles that are fixed between species by positive selection. In *Drosophila*, many genes are expected to influence the strength of TE epigenetic silencing and/or the associated side effects, such as those involved in the establishment of repressive marks at euchromatic TEs [13] or responsible for the maintenance and propagation of repressive epigenetic marks [64,65]. Interestingly, many of these genes are rapidly evolving, suggesting the action of positive selection (e.g. [66–69]). While functional tests are necessary to determine whether the evolution of these genes leads to enhanced or weakened TE silencing, several observations have hinted both types of changes are possible, such as reduced gene expression (weakening TE silencing, [24]) or increased gene copy number (enhancing silencing, e.g. [67]). An improved understanding of the functional impacts of between-species differences in genes involved in TE silencing may help further unravel the relative importance of various factors influencing the fixation probabilities of modifier alleles enhancing and weakening TE silencing.

Our observations revealed that, even if the epigenetic silencing of TEs is not a complete resolution of TE–host conflicts, the impacts on regulated transposition and the previously considered ‘curses’ of this conflict resolution could be a blessing, allowing stable containment of TEs, and have long-term benefits for host populations. On the other hand, whether modifier alleles that enhance or weaken TE silencing would be favoured over evolutionary time is more complex than a simple verbal model, and we need further estimates of key model parameters (e.g. rate of ectopic recombination) to resolve this question. It is worth noting that many genes involved in determining the strength of TE epigenetic silencing are highly pleiotropic, such as modulating the function of heterochromatin [65], maintaining genome stability [70] and influencing stem cell development [71]. The resolution of host–TE conflicts may thus have impacts beyond the immediately targeted selfish genetic elements and have global consequences on genome function and evolution.

## Data Availability

SLiM codes and scripts for analysing and plotting the simulation outcomes are available at https://github.com/YuhengHuang87/TE_silencing_simulations. Supplementary material is available online [72].
